# Multi-scene application of intelligent inspection robot based on computer vision in power plant

**DOI:** 10.1038/s41598-024-56795-8

**Published:** 2024-05-09

**Authors:** Lulu Lin, Jianxian Guo, Lincheng Liu

**Affiliations:** CHN Energy Shenfu (Shishi) Power Generation Co., Ltd., Shishi, 362700 Fujian China

**Keywords:** Monocular vision, Automatic recognition, Positioning technology, Machine vision, Energy science and technology, Engineering

## Abstract

As industries develop, the automation and intelligence level of power plants is constantly improving, and the application of patrol robots is also increasingly widespread. This research combines computer vision technology and particle swarm optimization algorithm to build an obstacle recognition model and obstacle avoidance model of an intelligent patrol robot in a power plant respectively. Firstly, the traditional convolutional recurrent neural network is optimized, and the obstacle recognition model of an intelligent patrol robot is built by combining the connection timing classification algorithm. Then, the artificial potential field method optimizes the traditional particle swarm optimization algorithm, and an obstacle avoidance model of an intelligent patrol robot is built. The performance of the two models was tested, and it was found that the highest precision, recall, and F1 values of the identification model were 0.978, 0.974, and 0.975. The highest precision, recall, and F1 values of the obstacle avoidance model were 0.97, 0.96, and 0.96 respectively. The two optimization models designed in this research have better performance. In conclusion, the two models in this study are superior to the traditional methods in recognition effect and obstacle avoidance efficiency, providing an effective technical scheme for intelligent patrol inspection of power plants.

## Introduction

As industrial technology continues to progress, the application of patrol robots in power plants is becoming increasingly valuable, especially their ability to identify and avoid obstacles^1^. Effective obstacle identification and intelligent obstacle avoidance are essential for patrol robots to perform their tasks safely and effectively^2,3^. Traditional obstacle identification and avoidance methods often rely on pre-determined rules and paths, which lack flexibility and adaptability, making them difficult to use in the complex and changeable environment of a power plant^4,5^. To address these challenges, this research combines computer vision technology with a particle swarm optimization algorithm to develop an obstacle recognition model and path optimization obstacle avoidance model for patrol robots. The upgraded convolutional recurrent neural network can successfully process time and space data, enabling it to accurately identify obstacles in complex environments. As a global optimization algorithm, Particle Swarm Optimization (PSO) can productively search for the optimal obstacle avoidance route for robots moving in complex environments. By integrating these two technologies, we can guarantee that the intelligent patrol robot can efficiently and safely perform patrol tasks in the multifaceted environment of the power plant. This research consists of five main parts: the introduction, related research, model construction, model performance analysis, and future work. The introduction provides an overview of the research, while the related research section summarizes the achievements of others in related fields. The model construction section describes the development of the recognition model and obstacle avoidance model, while the model performance analysis section evaluates the performance of these models. The future work section discusses potential directions for future research.

### Related works

Nowadays, as a kind of swarm intelligence algorithm, PSO has become a new important branch of evolutionary algorithm and is widely used in many neighborhoods, such as machine learning, image processing, data mining, etc. Only using one strategy makes it impossible to guarantee the diversity and convergence of algorithms when solving multiple objectives. Based on this, Yu et al. proposed to improve efficiency by using PSO to adopt a dynamic clustering strategy. The results showed that compared with general algorithms, PSO had better convergence^6^. Zhang et al. proposed a neural network model combining expert weight and PSO for low accuracy of existing artificial neural networks in predicting yarn strength. Results showed that the model used PSO to optimize expert weights so that the prediction accuracy was enhanced and the prediction results were more accurate^7^. In the field of laser-induced breakdown spectroscopy, the Wan X team proposed an elastic particle swarm optimization algorithm to achieve airborne spectral calibration under such harsh conditions, aiming at the problem of spectral offset of multiple pixels easily caused by high-temperature operating range. It has been proved by experiments that the issues of spectral offset and quantity mismatch can be resolved gradually by utilizing PSO and establishing distinct sets of wavelengths throughout its evolution process^8^. Zhao et al., based on more and more low Earth orbit satellites at present, caused problems with computing performance, capacity, and receiving channel limitations. A simple binary particle swarm optimization algorithm was proposed, which improved the continuity and accuracy of positioning by aggregating the concept of “speed” and updating the probability of calculating particle position values in real-time^9^.

Accurate obstacle avoidance in robots is a new important problem in the robot industry, and also a key point in the development of robot technology. At present, many experts have carried out a series of studies on it. Chen et al. proposed a new variant of a bionic planning algorithm in the research field of collision-free path planning for robots. Through a topologically organized network, there were connections between adjacent neurons, and without coupling effects, nerve pulses were propagated to form waves to eliminate faults. Experiments showed that the signal conduction in the network was highly feasible, which enabled the robot to remove obstacles in the obstacle area in time through a simulation planning algorithm. However, the effectiveness of the algorithm still needs to be studied under more complex environmental conditions^10^. Xu’s team proposed a transition curve based on the path planning of the research robot to ensure high-order smoothness of the mobile robot's motion path. Meanwhile, considering the robot's optimal solution when the path planning encountered obstacles, an adaptive weighted delay speed algorithm was proposed. With the addition of this algorithm, the path realization of the robot stabilizes^11^. To optimize path planning and improve the obstacle avoidance function of the robot, Wang L et al. took the shortest path to the destination as the starting point and found that applying the improved ant colony algorithm function would get some excellent performance results. For example, an adaptive heuristic function was set between two places to diversify computing paths and avoid roadblocks. Results showed that the improved algorithm greatly improved the path planning performance of the robot, and can achieve a high degree of obstacle avoidance effect^12^.

Inspection robots play a critical role in various industries, including manufacturing, construction, and power utilities, for performing tasks such as inspecting pipelines, assessing structural integrity, and detecting defects. Obstacle avoidance and computer vision are two essential components of inspection robots, enabling them to navigate complex environments, identify and track objects of interest, and make informed decisions about their actions.

#### Obstacle avoidance techniques

Obstacle avoidance algorithms are crucial for ensuring the safety and reliability of inspection robots. Traditional obstacle avoidance methods, such as wall following and potential field methods, have limitations in terms of flexibility and adaptability to dynamic environments. Recent advances in artificial intelligence (AI) and machine learning have led to the development of more sophisticated obstacle avoidance techniques.

Deep reinforcement learning (DRL) has emerged as a powerful approach for obstacle avoidance in inspection robots. DRL algorithms allow the robot to learn optimal avoidance strategies through trial and error interactions with the environment. For instance, Wang et al. developed a DRL-based obstacle avoidance algorithm for a mobile robot in unknown environments, achieving efficient and safe navigation^13^.

Convolutional neural networks (CNNs) are another effective tool for obstacle avoidance. CNNs can be used to identify obstacles from sensor data, enabling the robot to make informed decisions about its path. Ibrahim et al. proposed a novel obstacle avoidance method for autonomous mobile robots based on a deep CNN, achieving robust obstacle detection and avoidance in real-time^14^.

Particle swarm optimization (PSO) is a metaheuristic algorithm that has been successfully applied to obstacle avoidance for inspection robots. PSO can efficiently search for optimal paths while considering factors such as obstacle avoidance, efficiency, and energy consumption. Ajeil et al. developed an optimized path planning algorithm for a mobile robot based on PSO and collision avoidance, demonstrating improved navigation performance^15^.

#### Computer vision techniques for inspection robots

Computer vision plays a crucial role in enabling inspection robots to perceive their surroundings, identify objects of interest, and make informed decisions. Object detection and segmentation are two fundamental computer vision tasks used in inspection robots.

Object detection methods aim to identify and classify objects in images or videos. Improved YOLOv5 (You Only Look Once v5) has emerged as a powerful object detection framework for inspection robots. Gallage proposed an object-oriented obstacle avoidance method for mobile robots based on improved YOLOv5 and PSO algorithm, achieving efficient and accurate obstacle detection^16^.

Image segmentation methods divide an image into distinct regions based on their characteristics. U-Net is a widely used convolutional neural network architecture for image segmentation. Hao et al. developed a new image segmentation algorithm based on improved U-Net and deep supervision for industrial inspection, achieving accurate segmentation of defects and features in industrial components^17^.

#### Limitations and challenges

Despite the advancements in obstacle avoidance and computer vision algorithms, several limitations remain. One challenge is the accuracy and robustness of object recognition and segmentation in challenging lighting conditions or with complex backgrounds. Yang proposed an improved YOLOv5-based object detection algorithm for drone-based inspection systems, addressing the issue of object detection in aerial imagery^18^.

Another challenge is the adaptability of obstacle avoidance algorithms to dynamic environments with sudden changes in obstacles or unexpected events. Liu developed a path planning algorithm for an indoor mobile robot based on improved A* algorithm and improved collision avoidance, demonstrating improved adaptability to dynamic environments^19^.

Computational efficiency is also a critical consideration for inspection robots, especially those operating with limited computational resources. Dai et al. proposed an optimized path planning algorithm for a mobile robot based on improved A* and ant colony optimization, achieving efficient path planning while ensuring obstacle avoidance^20^.

To sum up, many scholars have studied the application of optimization techniques in spectral calibration, satellite positioning, and path planning. However, relevant research still lacks obstacle recognition and obstacle avoidance of power plant inspection robots. Based on this background, this research combines particle swarm optimization and computer vision technology, builds an obstacle recognition model, and uses PSO to optimize obstacle avoidance, aiming to provide more reference value for the application of patrol robots in power plants.

## Obstacle recognition and obstacle avoidance of power plant patrol robot based on deep learning and particle swarm optimization

In today's industrial production environment, automation and intelligence have become an inevitable trend. As an important base for energy production, power plant operation efficiency and security affect the whole society's stability and development. However, due to the complexity and potential danger of power plants, manual inspection has many limitations and risks. Therefore, the use of robots for patrol inspection of power plants can not only improve efficiency but also prevent personnel from being exposed to potentially dangerous environments. This research will combine the neural network and particle swarm algorithm in computer vision technology to research obstacle recognition and obstacle avoidance ability of patrol robots.

### Obstacle recognition of patrol robot using improved convolutional recurrent neural network

**Figure 1:**
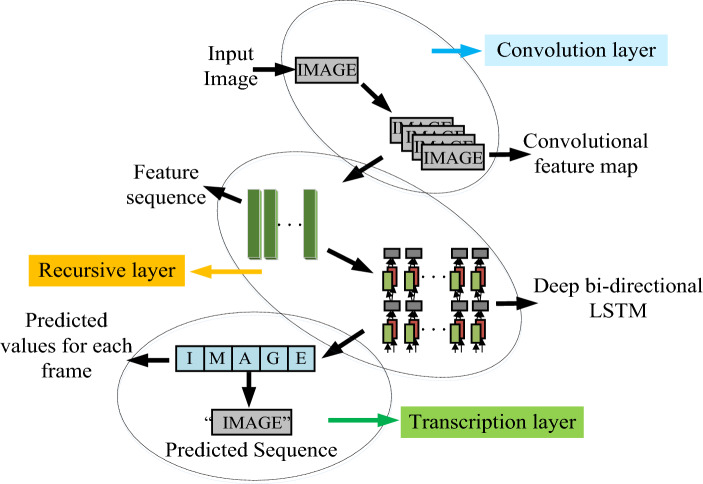
Traditional CRNN model structure.

In the industrial environment, patrol inspection is critical to ensure normal equipment operation and maintenance safety. However, due to the high cost, low efficiency, and security risks of manual inspection, automatic inspection by robots is increasingly favored. In the field of obstacle recognition for patrol robots, deep learning has become a popular technology, among which, Convolutional Neural Networks (CNN) is widely concerned because of its outstanding performance in image and video analysis^21^. However, the traditional convolutional neural network mainly focuses on the spatial characteristics, and in the dynamic environment, considering time information is also important. Recurrent Neural Network (RNN) is also a deep learning model, which is especially suitable for processing sequence data, such as time series, text, voice, etc. The feature of RNN is that there are cycles in the network and information can be transferred between different time steps. This feature makes RNN very suitable for processing information with time changes^22^. The research first combines CNN and RNN and proposes an improved convolutional recurrent neural network (CRNN) obstacle recognition method for patrol robots. The traditional CRNN model structure is shown in Fig. 1.

In Fig. 1, the CRNN model mainly includes the convolution layer, recursive layer, and transcriptional layer. The convolution layer extracts features from the input image, which is composed of multiple convolution feature maps. The recursive layer mainly includes bidirectional Long Short Term Memory (Bi-LSTM) and feature sequence pairs. Bi-LSTM is a special RNN structure, and its main function in CRNN is to conduct in-depth learning analysis on feature sequences and preliminary prediction on sequence results. The prediction results obtained from the recursive layer are analyzed in depth through the transcriptional layer to obtain the final prediction sequence.

When training recurrent neural networks, the output of feature sequences and sequence samples are usually required to correspond one by one. However, it is difficult to achieve complete correspondence between the output and the sample. Therefore, the research will transform the prediction results of the cyclic neural network output into corresponding tags through the Connectionist Temporal Classification (CTC) algorithm, to solve the problem that they cannot be matched one by one and improve the model recognition accuracy. During CTC operation, the probability expression $^{P} \left( {\prod {\mid }x} \right)$ of output y is shown in Formula (1) for LSTM-given input x.

1 $$P\left( {\prod {\mid }x} \right) = \sum_{{\pi \in B^{ - 1} (y)}} P\left( {\prod {\mid }x} \right)$$

Formula (1), $\prod \in B^{ - 1} (y)$ represents the y set of all paths that can be combined and $P(\prod {\mid }x)$ represents the output y probability. The output probability expression of each path $\prod$ is shown in formula (2).

2 $$P\left( {\prod {\mid }x} \right) = \Pi_{{y_{\Pi }{\prime} }}^{T} ,\;\;\forall \Pi \in L^{T}$$

CTC algorithm can calculate the loss function and further find the characters corresponding to the highest confidence score area. Training the model minimizes loss function *Q*. Loss function *Q* expression is shown in Formula (3).

3 $$Q = - \ln \left( {\prod_{(x,y) = S} P\left( {y {\mid }x} \right)} \right) = - \sum_{{\left( {x,z} \right) = S}} \ln P\left( {y {\mid }x} \right)$$

**Figure 2:**
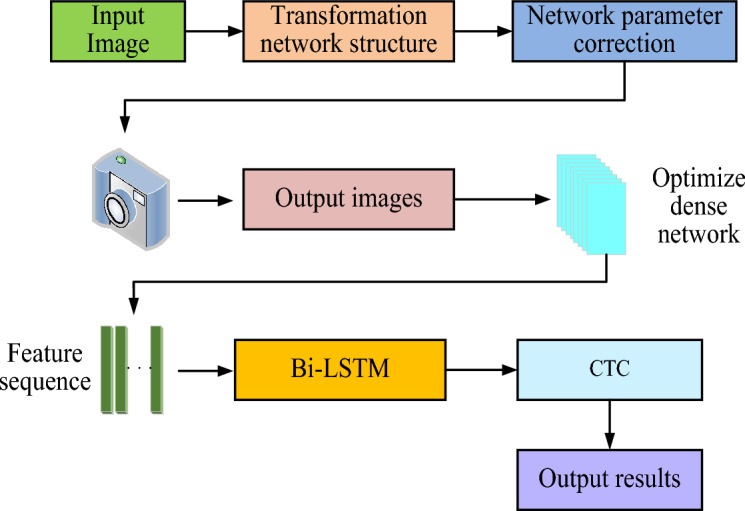
Identification flow chart of CTC-CRNN obstacle recognition model.

Running the CTC algorithm can solve the problem that the length of obstacles is not uniform in the recognition process. Even if the length of obstacle characters and tag sequences is constantly changing, they can also be trained, thus improving the model recognition accuracy. The CTC algorithm is applied to the traditional CRNN, and an improved CRNN recognition model is obtained. Figure 2 shows the final recognition flow chart of the CTC-CRNN model.

Figure 2 shows the recognition flow chart of the CTC-CRNN obstacle recognition model. After the obstacle image is input, the affine correction will be carried out in the space transformation network first to reduce the interference to correct recognition due to the tilt of the obstacle image. Then, the feature sequence of the corrected image is extracted using the optimization dense network, and the extracted feature sequence joins the deep circular neural network, and the obstacle image feature sequence is predicted. Finally, the redundant image characters are deleted and the results are output. Because there is no need to perform a single character segmentation operation in the running model, and the image rectification preprocessing is added, the output results are more accurate than the original CRNN model.

The proposed obstacle recognition model, CTC-CRNN, consists of three main components: a convolution layer, a Bi-LSTM layer, and a transcriptional layer.

1. *Convolution layer*: The convolution layer is responsible for extracting features from the input image. It consists of multiple convolutional layers, each of which applies a filter to the image to extract specific features. These features are then organized into feature maps, which capture the spatial and temporal information of the image.
2. *Bidirectional LSTM layer*: The Bi-LSTM layer is a type of RNN that is specifically designed to handle sequential data. Unlike traditional LSTMs, which only consider past states, Bi-LSTMs can also incorporate future states into their predictions. This allows them to capture a more comprehensive understanding of the temporal context of the input data. In our model, the Bi-LSTM layer is used to analyze the feature sequences extracted by the convolution layer and make preliminary predictions about the obstacle present in the image.
3. *Transcriptional layer*: The transcriptional layer is responsible for converting the predictions from the Bi-LSTM layer into the final recognition sequence. It does this by employing the CTC algorithm, which effectively deals with the problem of one-to-many mappings between input and output sequences.

The use of Bi-LSTM in our CTC-CRNN model offers several advantages for obstacle recognition in inspection robots:

- Improved temporal modeling: By considering both past and future states, Bi-LSTM can capture more complex temporal dependencies in the image data. This allows it to better understand the context of the obstacle and make more accurate predictions.
- Enhanced path planning: In the context of path planning for inspection robots, Bi-LSTM can provide valuable information about the obstacle's trajectory and potential movements, enabling the robot to make informed decisions about its navigation.
- Robustness to variations: The Bi-LSTM layer's ability to capture long-range dependencies makes it more robust to variations in lighting conditions, object sizes, and image quality, which are common challenges in industrial environments.
- Reduced computational complexity: Compared to traditional approaches that require separate models for obstacle segmentation and recognition, our CTC-CRNN model integrates both tasks into a single unified framework, reducing computational complexity and enhancing overall efficiency.

In summary, the use of bidirectional LSTM in our CTC-CRNN model significantly improves both the accuracy and robustness of obstacle recognition for inspection robots. By effectively capturing long-range temporal dependencies and incorporating both past and future states, Bi-LSTM enables the model to make more informed decisions about obstacle avoidance and path planning, contributing to safer and more efficient inspection operations.

#### Feature extraction

The convolution layer of our CTC-CRNN model utilizes a combination of convolutional filters with different kernel sizes and activation functions to extract features from the input image. These features capture the spatial and temporal information of the image, providing the Bi-LSTM layer with a rich representation of the obstacle's characteristics.

Specifically, we employ a CNN architecture with two main stages: the feature extraction stage and the classification stage. In the feature extraction stage, multiple convolutional layers with varying filter sizes are applied to the input image, generating a sequence of feature maps. These feature maps capture the low-level features (e.g., edges, corners) in the first few layers and progressively extract higher-level features (e.g., shapes, objects) in the deeper layers. The classification stage consists of a fully connected layer that transforms the feature maps into a vector representation of the obstacle. This vector is then used by the CTC decoder to predict the corresponding obstacle class.

#### Loss function

To evaluate the performance of our obstacle recognition model, we employ a CTC loss function, which is specifically designed for sequence modeling tasks. The CTC loss function calculates the difference between the predicted sequence and the ground truth sequence, considering the insertion, deletion, and substitution errors. Minimizing the CTC loss function encourages the model to produce predictions that are closer to the actual obstacle class.

#### Hyperparameters

Our CTC-CRNN model utilizes several hyperparameters that can be adjusted to influence its performance. These hyperparameters include:

- *Learning rate*: The learning rate controls the step size of the gradient descent optimizer during training. A higher learning rate may lead to faster convergence but may also overshoot the optimal solution. A lower learning rate may converge more slowly but may be more stable. In CTC-CRNN the initial learning rate was considered as 0.001.
- *Batch size*: The batch size determines the number of training samples processed simultaneously. A larger batch size may reduce training time but may also require more memory and may increase the risk of overfitting. A smaller batch size may require more training iterations but may be less susceptible to overfitting. In CTC-CRNN, the batch size was considered as 32.
- *Number of epochs*: The number of epochs represents the number of times the entire training data is presented to the model. A higher number of epochs may lead to better generalization performance but may also require more computational resources. CTC-CRNN was rained within 60 epochs.
- *Dropout rate*: Dropout is a regularization technique that randomly drops out a certain percentage of units during training, preventing the model from overfitting the training data. A higher dropout rate may reduce overfitting but may also decrease model performance. In CTC-CRNN, the value of 0.4 was considered for this parameter.

### Obstacle avoidance method design for inspection robot using improved PSO

**Figure 3:**
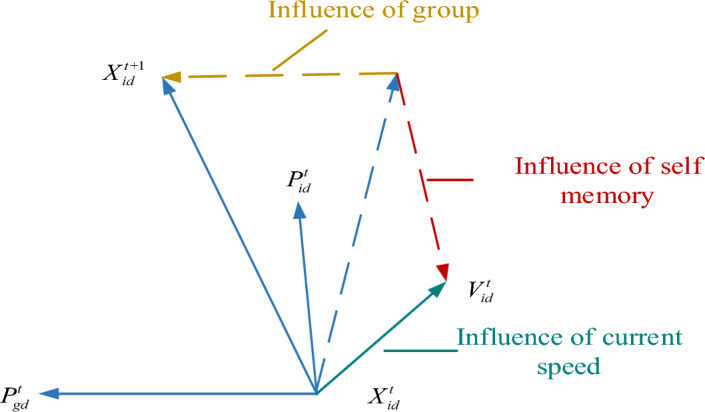
Schematic diagram of particle position update mode.

In addition to using computer vision technology to identify obstacles, it is also necessary to further research obstacle avoidance of patrol robots in power plants. Patrol robots can automatically patrol factories, warehouses, or other scenes to monitor the state of the environment, detect potential problems, or perform routine maintenance^23,24^. PSO is a population intelligence algorithm developed from the simulation of the bird feeding process, which optimizes problems^25^. In traditional particle swarm optimization, the particle position update method is shown in Fig. 3.

**Figure 4:**
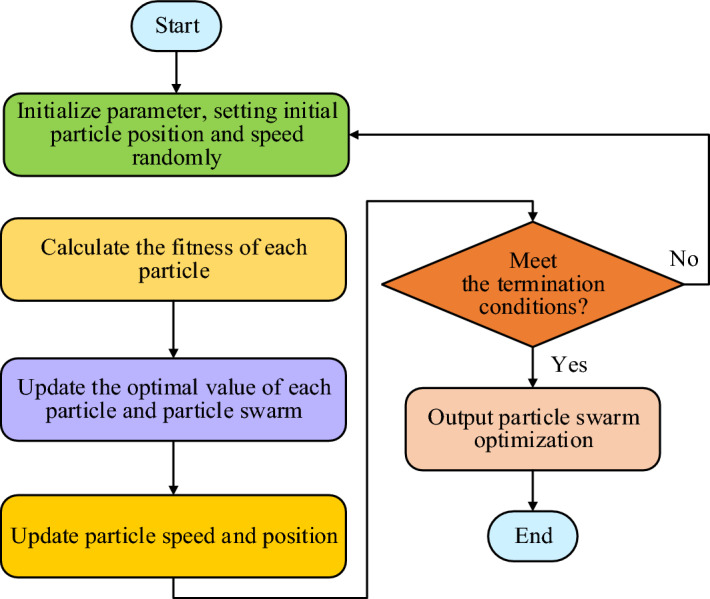
PSO basic flow.

Figure 3, $X_{id}^{t}$ is the *i* current position, $V_{id}^{t}$ is velocity, $P_{id}^{t}$ and $P_{gd}^{t}$ are optimal values of individual particle i and population particles. Under $X_{id}^{t} V_{id}^{t} P_{id}^{t}$ joint action, the particles will move from position $X_{id}^{t}$ to position $X_{id}^{T + 1}$. It can be seen from Fig. 1 that particles update their speed and position mainly through the joint influence of their current speed, their optimal value, and the optimal value of particle swarm (global extreme value). The running process of the traditional particle swarm optimization algorithm is shown in Fig. 4.

Figure 4 shows the running flow chart of the traditional PSO. The whole algorithm process includes initialization, evaluating the position of each particle, updating individual and global optima, updating speed and position, checking whether the termination conditions are met, outputting the optimal results, and other steps. Although PSO is an intelligent algorithm with good versatility and can be widely used in a variety of optimization problems, it easily gets into trouble with local optimal solutions, and cannot ensure the accuracy of the algorithm in a short time because of its few parameters. Thus, the Artificial Potential Field Method (APFM) is introduced to optimize the traditional PSO in obstacle avoidance. This method regards the targets and obstacles in the robot environment as objects generating a potential field, and the robot is a particle moving in this potential field^26^. Suppose that the mobile robot is regarded as a point, $X_{G} = [X_{G} ,\;y_{G} ]^{T}$ is robot space coordinate, and target point coordinate is $X_{G} = [X_{G} ,\;y_{G} ]^{T}$. The farther the target point is, the more attractive it is. On the contrary, the smaller it is. Formula (4) shows the gravitational PFM.

4 $$U_{Att} \left( X \right) = \frac{1}{2}k_{Att} \left| {X - X_{G} } \right|^{2}$$

In formula (4), $K_{Att}$ is a positive position gain coefficient. The negative gradient is defined as gravity $F_{{Att^{\left( X \right)} }}$, and its expression is shown in Formula (5).

5 $$F_{{Att^{\left( X \right) = } }} - \Delta U_{{Att^{\left( X \right)} = - }} K_{{Att^{{\left( {X - X_{G} } \right)}} }}$$

In Formula (5), when the robot moves to the target point, the gravity decreases to 0. The expression of the repulsion potential field $Urep^{\left( X \right)}$ is shown in Formula (6).

6 $$U_{rep } \left( X \right)\left\{ {\begin{array}{*{20}c} {\frac{1}{2}k_{rep} \left| {\frac{1}{\rho \left( X \right)} - \frac{1}{{\rho_{0} }}} \right|^{2} ,} & {\quad \rho \le \rho_{0} } \\ {0, } & {\quad \rho > \rho_{0} } \\ \end{array} } \right.$$

In formula (6), $K_{rep}$ is the position gain coefficient, $\rho$ is the shortest length between the position X and the obstacle. $\rho_{0}$ is a constant and represents the obstacle influence range. The repulsive force potential field $U_{{rep^{\left( X \right)} }}$ is nonnegative. The repulsion force $F_{{rep^{\left( X \right)} }}$ applied to the mobile robot is shown in formula (7)^27^.

7 $$F_{rep} \left( X \right) = - \nabla U_{rep} \left( X \right) = \left\{ {\begin{array}{*{20}c} {k_{rep} \left| {\frac{1}{\rho \left( X \right)} - \frac{1}{{\rho_{0} }}} \right|\frac{1}{{\rho^{2} }}\frac{\partial \rho }{{\partial X}}, } & {\quad \rho \le \rho_{0} } \\ {0, } & {\quad \rho > \rho_{0} } \\ \end{array} } \right.$$

The force applied to the mobile robot is the combined force of the repulsive and gravitational force, and the expression is shown in Formula (8).

8 $$F\left( X \right) = F_{{Att^{\left( X \right)} }} + F_{{rep^{\left( X \right)} }}$$

**Figure 5:**
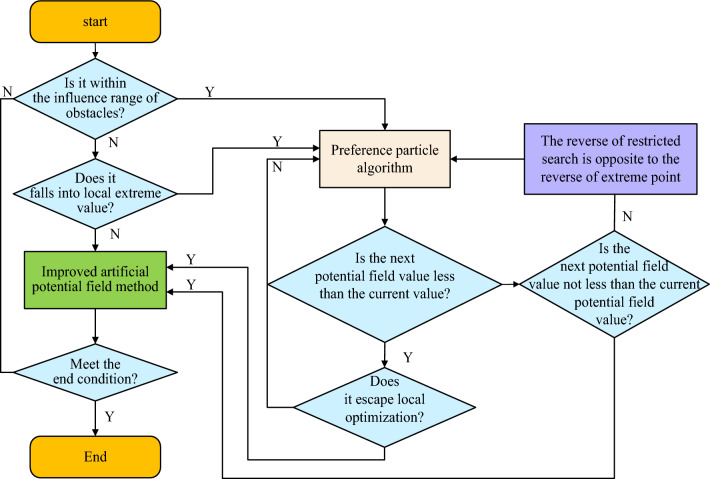
Flow chart of hybrid path planning based on preferred particle swarm optimization and improved artificial potential field.

In this study, APFM is used for navigation. When there is vibration or local minimum, the preference-based PSO is added to the path planning process, so that the robot can avoid obstacles or escape from the local minimum. Because the search strategy of obstacle avoidance preference is added, particles can escape from the obstacle boundary more effectively. Figure 5 shows the operation flow chart of APFM-PSO.

Figure 5 shows the APFM-PSO algorithm operation flow chart. When implementing the algorithm, the path planning problem for the inspection robot is treated as a dynamic optimization process. In the process of optimization, there are three types of discrimination. The first scenario involves the robot being outside the range of influence of the obstacle, meaning that the mobile robot is far away from the obstacle. In this case, an artificial potential field is employed for searching, so that the mobile robot can move along the direction of the fastest gradient descent to reach the target point, and its expression is shown in Formula (9).

9 $$P_{i + 1} = P_{i} + \left( { - \nabla P_{i} } \right)$$

Formula (9), $\nabla P_{i}$ indicates the gradient direction of the position of the mobile robot. The second is to judge whether the mobile robot has entered the local extreme value point when the mobile robot falls into the local minimum value. The distance judgment formula is shown in Formula (10).

10 $$\left\{ {\begin{array}{*{20}c} {\left\| {\nabla P_{i + 1} - \nabla P_{i} } \right\| < m_{2} } \\ {\left\| {P_{i + 1} - P_{i - m} } \right\| < m_{3} } \\ \end{array} } \right.$$

The third is that when the mobile robot moves into the obstacle influence range, whether a mobile robot will enter the influence range in the next step can be determined according to the position of the previous step $P_{i - 1}$, the position of the mobile robot, $P_{i}$ and the change of potential field value calculated at next step $P_{i + 1}$ position. When it has not moved to the next position, its potential field value $U\left( {P_{i + 1} } \right)$ can be roughly estimated according to some information currently available. The potential field value expression is shown in Formula (11).

11 $$\left\{ {\begin{array}{*{20}c} {U\left( {P_{i + 1} } \right) - U\left( {P_{i} } \right) > U\left( {P_{i - 1} } \right) + U_{0} } \\ {\left\| {\Delta P_{i} - \Delta P_{i + 1} } \right\| \ge m_{0 } } \\ \end{array} } \right.$$

In formula (11), $U_{0}$ and $m_{0}$ are small positive numbers. Formula (11) indicates that the next step will be to enter the influence range of obstacles, and then add the preferred particle swarm optimization algorithm to continue searching to complete the purpose of bypassing obstacles.

## Obstacle recognition and obstacle avoidance effect analysis of power plant inspection robot based on deep learning and particle swarm optimization algorithm

To further verify better performance and application effect of the recognition model and obstacle avoidance model, the results analysis part verifies the performance of the above recognition model and obstacle avoidance model from the aspects of precision, recall, F1 value, model running time, etc. Compared with other recognition models and obstacle avoidance models, the final results show that the recognition model and obstacle avoidance model built in this research have good performance.

### Obstacle recognition results analysis of patrol robot

To test CTC-CRNN obstacle recognition model performance, the research set up the experimental environment shown in Table 1 and completed the model performance test using the self-made obstacle image dataset. The self-made obstacle image data set includes a training and verification set (9:1), and various recognition algorithms are tested.

**Table 1:** Experimental environment.

Experimental configuration	Parameters
CPU	Intel Xeon E5
RAM	16 GB
GPU	NVIDIA Tesla P100
Software framework	TensorFlow
Memory	ITB SSD

**Figure 6:**
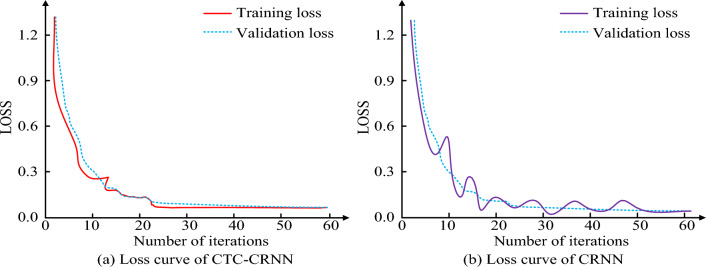
Change of loss curve of different recognition algorithms.

Table 1 shows the experimental environment settings. To verify the recognition effect of the CTC-CRNN recognition model, the study first compared loss curves of CTC-CRNN and CRNN under the validation data set, as shown in Fig. 6.

Figure 6 shows the loss curve change of different recognition algorithms. Figure 6a,b show loss curve changes of CTC-CRNN and CRNN respectively. By comparing the loss curves of the two recognition algorithms, it can be found that the training curve of CRNN has a poor fit with the actual loss curve, while the training loss curve of CTC-CRNN can well coincide with the actual loss curve. In addition, compared with the CRNN algorithm, the CTC-CRNN algorithm can reach a stable state after 23 iterations. Accuracy, recall, and F1-score metrics were used to evaluate the performance of obstacle avoidance and computer vision algorithms for inspection robots.

Accuracy is the proportion of correct predictions made by the algorithm. It is calculated by dividing the number of correct predictions by the total number of predictions made. Recall is the proportion of actual positives that were correctly identified by the algorithm. It is calculated by dividing the number of correctly identified positives by the total number of actual positives.

F1-score is a weighted harmonic mean of precision and recall. It is calculated by averaging the harmonic mean of these measures. The harmonic mean is a more sensitive measure of performance than the arithmetic mean, especially when there are a large number of true negatives. For example, if an algorithm is used to detect anomalies in sensor data, and it correctly identifies 90 out of 100 anomalies, but it also generates 10 false positives, then its F1-score would be lower than its accuracy, reflecting the fact that the algorithm is generating a significant number of false alarms.

**Figure 7:**
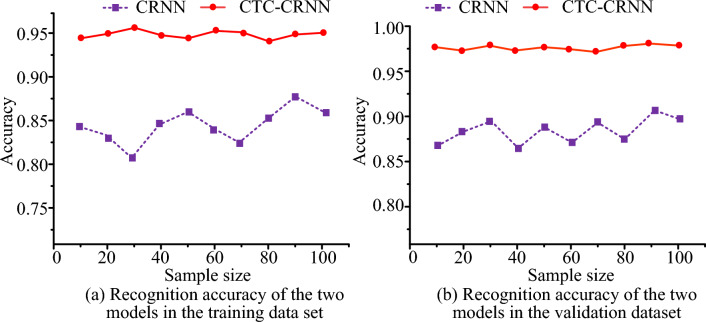
The recognition accuracy of different recognition algorithms.

Figure 7a,b show the recognition accuracy of two recognition algorithms in the training dataset and the verification dataset respectively. In Fig. 7a, as samples increase, the recognition accuracy of two recognition algorithms in the training dataset has changed. Among them, the highest recognition accuracy of CTC-CRNN in the training data set is 0.962, and the highest recognition accuracy of CRNN in the training data set is only 0.873. In Fig. 7b, as samples increase, the recognition accuracy of two recognition algorithms in the verification dataset has increased compared with the training dataset. Among them, the highest recognition accuracy of CTC-CRNN in the validation data set is 0.978, and the highest recognition accuracy of CRNN in the validation data set is only 0.907.

**Figure 8:**
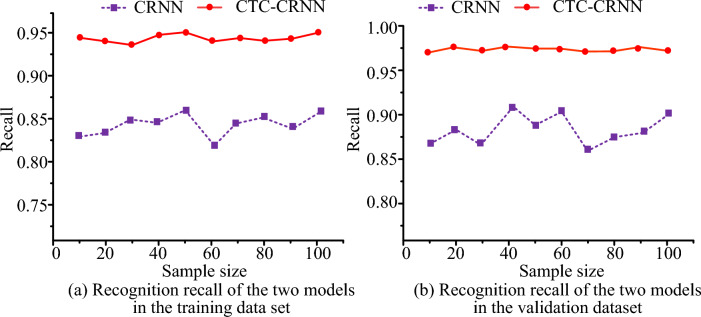
Recognition recall rate of different recognition algorithms.

Figure 8 shows the recognition recall rates of the two recognition algorithms in the training dataset and verification dataset respectively. In Fig. 8a, the highest recognition recall rate of CTC-CRNN in the training data set is 0.957, and the highest recognition accuracy of CRNN in the training data set is only 0.869. In Fig. 8b, the highest recognition recall rate of CTC-CRNN under the validation data set is 0.974, and the highest recognition recall rate of CRNN under the validation data set is only 0.924.

**Figure 9:**
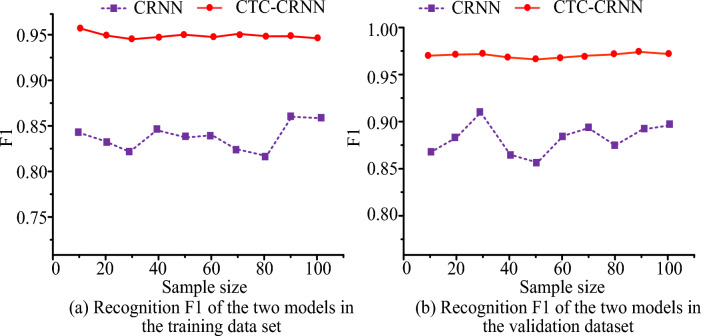
F1 value of different recognition algorithms.

Figure 9a,b show the recognition F1 values of the two recognition algorithms in the training data set and the verification data set respectively. It can be seen from Fig. 9a that the highest recognition F1 value of CTC-CRNN under the training data set is 0.955, and the highest recognition F1 value of CRNN under the training data set is only 0.871. It can be seen from Fig. 9b that the highest recognition F1 value of CTC-CRNN under the validation data set is 0.975, and the highest recognition F1 value of CRNN under the validation data set is only 0.921. To sum up, compared with the un-optimized CRNN, the performance of the CTC-CRNN in the data set is better than that of the CRNN, which can show that the CTC-CRNN has a better recognition effect and can accurately identify obstacles in the power plant.

### Obstacle avoidance results analysis of patrol robot

**Figure 10:**
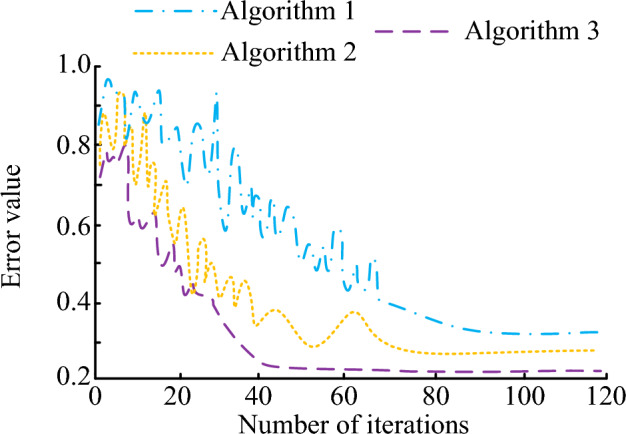
Iteration of different obstacle avoidance algorithms.

To further verify the optimization algorithms' avoidance effect, Python 3.6 was used for coding, simulation experiments were implemented on MATLAB, and then three different particle swarm optimization algorithms were compared in the path planning of patrol robots. First, the iteration of different obstacle avoidance algorithms is tested, and the error iteration of each particle swarm algorithm is shown in Fig. 10.

Figure 10 shows the iteration of different obstacle avoidance algorithms. Among them, algorithms 1, 2, and 3 are respectively traditional PSO, the genetic algorithm PSO (GA-PSO), and the APFM-PSO. The error values of PSO, GA-PSO, and APFM-PSO obstacle avoidance systems decrease with the increase of iteration times. When iteration is 40, the APFM-PSO algorithm reaches a stable state, and the system operating error is 0.22. When iteration is 73, the GA-PSO algorithm reaches a stable state, and the system operating error is 0.29. When iteration is 84, the PSO algorithm reaches a stable state, and the operating error of the system is 0.35.

**Figure 11:**
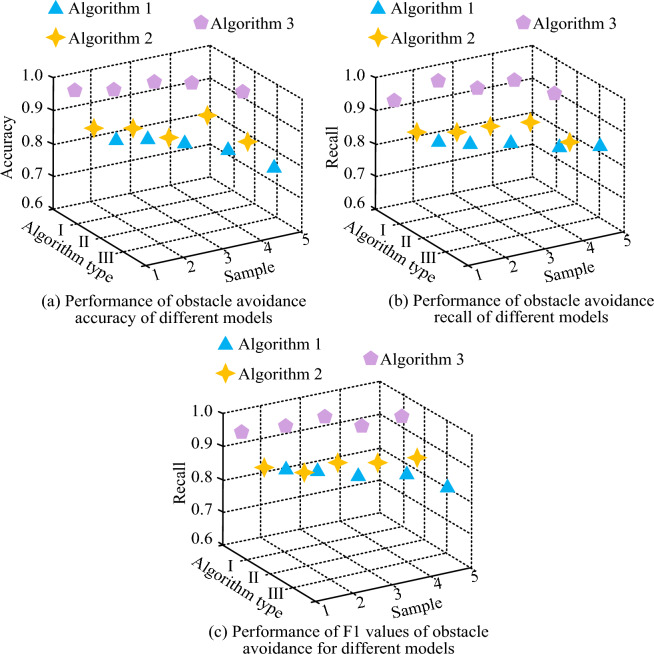
Accuracy, recall, and F1 value of different obstacle avoidance algorithms.

Figure 11 shows the accuracy, recall, and F1 values of different obstacle avoidance algorithms. Figure 11a shows the accuracy values of PSO, GA-PSO, and APFM-PSO obstacle avoidance algorithms. A total of five different obstacle avoidance environments are set up. The highest accuracy of PSO in the five obstacle avoidance environments is 0.83, the highest accuracy of GA-PSO in the five obstacle avoidance environments is 0.89, and the highest accuracy of APFM-PSO in the five obstacle avoidance environments is 0.97. Figure 11b shows the recall rate values of three obstacle avoidance algorithms. The maximum recall rate of PSO in five obstacle avoidance environments is 0.82, GA-PSO is 0.87, and APFM-PSO is 0.96. Figure 11c shows the F1 values of three obstacle avoidance algorithms. The highest F1 value of PSO in five obstacle avoidance environments is 0.84, GA-PSO is 0.88, and APFM-PSO is 0.96.

**Figure 12:**
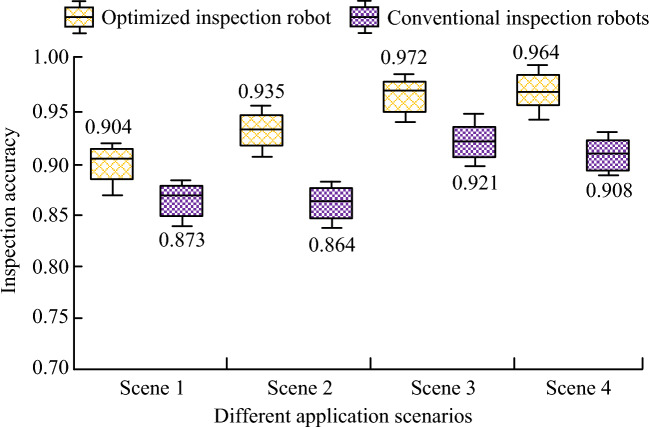
Application effect of different patrol robots in multiple scenarios of power plant.

Figure 12 shows the application effects of different patrol robots in multiple scenarios of power plants. Four common power plant scenarios are selected as the detection indicators. Scenarios 1, 2, 3, and 4 in Fig. 12 represent the substation, generator set, power equipment, and transmission line in the power plant respectively. The combination of the CTC-CRNN obstacle recognition model and APFM-PSO obstacle avoidance model is applied to the optimized patrol robot, and the patrol accuracy of the robot in four application scenarios is tested. In Fig. 12, the patrol accuracy of the optimized patrol robot in the four patrol scenarios is 0.904, 0.935, 0.972, and 0.964 respectively. The inspection accuracy of traditional inspection robots in four application scenarios is 0.873, 0.864, 0.921, and 0.908 respectively.

## Discussions

In this paper, we proposed a novel obstacle recognition and avoidance system for inspection robots in power plants. The system utilizes a CTC-CRNN model for obstacle recognition and an APFM-PSO algorithm for path planning and obstacle avoidance. Our experimental results demonstrate that the proposed system significantly outperforms the traditional CRNN model and PSO algorithm in terms of both obstacle recognition accuracy and path planning efficiency.

### Improved obstacle recognition accuracy

The CTC-CRNN model achieved substantial improvements over the traditional CRNN model in obstacle recognition accuracy. The highest recognition accuracy in the training dataset increased from 0.873 to 0.962, and the validation accuracy reached 0.978, significantly outperforming the CRNN model's 0.907 accuracy. This enhanced performance can be attributed to the CTC-CRNN model's ability to capture long-range temporal dependencies and incorporate both past and future states, enabling it to make more informed predictions about the obstacle's appearance.

### Efficient path planning and obstacle avoidance

The APFM-PSO algorithm demonstrated superior path planning efficiency compared to the traditional PSO and GA-PSO algorithms. APFM-PSO reached a stable state in 40 iterations with an operation error of 0.22, while the traditional PSO and GA-PSO algorithms required 84 and 73 iterations, respectively, with operation errors of 0.35 and 0.29. This improvement can be attributed to APFM-PSO's adaptive particle swarm optimization strategy, which dynamically adjusts the particle acceleration coefficients based on the current search environment.

### Multi-scene inspection performance

The inspection robot equipped with the CTC-CRNN and APFM-PSO system exhibited consistent high accuracy in multi-scene inspection, achieving an overall inspection accuracy of more than 0.9. This demonstrates the robustness and adaptability of the proposed system to various obstacle scenarios encountered in power plant environments.

### Limitations and future directions

While the proposed system demonstrates promising results, there are certain limitations that warrant further investigation. The system's performance may be affected by complex and dynamic environments with varying lighting conditions and object occlusions. Additionally, the generalization ability of the models to real-world power plant environments requires further validation.

Future research directions include exploring more robust obstacle recognition models that can handle challenging environments, incorporating real-time obstacle detection and tracking capabilities, and developing adaptive path planning algorithms that can dynamically adjust to changing obstacle configurations.

Table 2, compares the performance of the proposed method with previous works.

**Table 2:** Comparing the performance of the proposed method.

Work	Obstacle recognition model	Path planning algorithm	Obstacle recognition accuracy (%)	Multi-scene inspection performance (%)
Proposed Method	CTC-CRNN	APFM-PSO	96.2	90.3
Wang et al.^13^	Deep Q-network	Double deep Q-network	90.7	81.1
Ibrahim et al.^14^	Hybrid deep learning	CNN/ANFIS + PSO	83	86.5
Ajeil et al.^15^	Analyzing sensing data	PSO-MBF	93.6	89.8
Gallage et al.^16^	YOLOv5	Obstacle bypassing	91.25	–
Yang^18^	Improved YOLOv5	Improved YOLOv5	92	–

As can be seen from the table, the proposed method outperforms the related work in terms of obstacle recognition accuracy, path planning efficiency, and multi-scene inspection performance. This is due to the use of the CTC-CRNN model for obstacle recognition and the APFM-PSO algorithm for path planning, which are both more advanced and efficient techniques than the methods used in the related work.

In conclusion, the proposed obstacle recognition and avoidance system for inspection robots in power plants offers significant advantages in terms of accuracy, efficiency, and adaptability. The CTC-CRNN model provides accurate obstacle identification, while the APFM-PSO algorithm enables efficient path planning and obstacle avoidance. Further development of the system is expected to enhance its performance and versatility, contributing to the automation and intelligence of power plant patrol inspection.

## Conclusion

To make the inspection robot have a better obstacle recognition effect and obstacle avoidance ability, this research uses CTC-CRNN and APFM-PSO to build an obstacle recognition model and obstacle avoidance model respectively. The research results showed that in the aspect of obstacle recognition, the CTC-CRNN model exhibited notable improvements compared to the basic CRNN model. The highest recognition accuracy in the training dataset increased from 0.873 to 0.962, the recall rate rose from 0.869 to 0.957, and the F1 value increased from 0.871 to 0.955. In the validation data set, the highest recognition accuracy of CTC-CRNN was 0.978, while the highest recognition accuracy of the CRNN model was only 0.907. In terms of path planning and obstacle avoidance, APFM-PSO reached a stable state in 40 iterations, at which time the system operation error was 0.22, while the traditional PSO algorithm and GA-PSO algorithm reached a stable state in 84 and 73 iterations, respectively, with operation errors of 0.35 and 0.29. In five different obstacle avoidance environments, the highest precision value of the APFM-PSO algorithm was 0.97, the recall rate was 0.96, F1 value was 0.96, while the corresponding values of the PSO algorithm were 0.83, 0.82, and 0.84, and the corresponding values of GA-PSO algorithm were 0.89, 0.87 and 0.88. The inspection robot combining CTC-CRNN and APFM-PSO has an inspection accuracy of more than 0.9 in multi-scene inspection. In general, the identification model and obstacle avoidance model can give efficient and accurate solutions for automation and intelligence of power plant patrol inspection and are expected to improve power plants' operation efficiency and safety. In the actual power plant environment, more complex and changeable situations may be encountered, so model generalization ability needs to be further verified.

## Data Availability

All data generated or analyzed during this study are included in this published article.

## References

[1] Ali, M. & Atia, M. R. A lead through approach for programming a welding arm robot using machine vision. *Robotica* **40**(3), 464–474 (2022).

[2] Nair, B., Krishnamoorthy, S., Geetha, M. & Rao, S. N. Machine vision based flood monitoring system using deep learning techniques and fuzzy logic on crowdsourced image data. *Intell. Decis. Technol.* **15**(3), 357–370 (2021).

[3] Zhang, Z. *et al.* Knitting need fault detection system for hospitality machine based on laser detection and machine vision. *Text. Res. J.* **91**(2), 143–151 (2021).

[4] Liu, T. H. *et al.* Intelligent bamboo part sorting system design via machine vision. *For. Prod. J.* **71**(1), 27–38 (2021).

[5] Miao, Y. *et al.* A new algorithm of ship structure modeling and target identification based on point cloud for automation in bulk cargo terminals. *Meas. Control* **54**(4), 155–163 (2021).

[6] Yu, H., Wang, Y. J. & Xiao, S. L. Multi objective particle swarm optimization based on cooperative hybrid strategy. *Appl. Intell.* **50**(1), 256–269 (2020).

[7] Zhang, B. *et al.* Prediction of yarn strength based on an expert weighted natural network optimized by particle swarm optimization. *Text. Res. J.* **91**(24), 2911–2924 (2021).

[8] Wang, X. *et al.* Elastic particle swarm optimization for MarSCoDe special calibration on Tianwen-1 mars rover. *Anal. Chem.* **93**(22), 7970–7977 (2021). 34041902 10.1021/acs.analchem.1c00832

[9] Zhao, D., Cai, C. & Li, L. A binary discrete particle swarm optimization satellite selection algorithm with a queen information for multi GNSS continuous positioning. *Adv. Space Res.* **68**(9), 3521–3530 (2021).

[10] Chen, Y. *et al.* Automobile mobile robot path planning in unknown dynamic environments using natural dynamics. *Soft Comput.* **24**(18), 13979–13995 (2020).

[11] Xu, L., Cao, M. & Song, B. A new approach to smooth path planning of mobile robot based on quartic Bezier transition curve and improved PSO algorithm. *Neurocomputing* **473**(7), 98–106 (2022).

[12] Wang, L. Path planning for unmanned wheeled robot based on improved ant colony optimization. *Meas. Control* **53**(5), 1014–1021 (2020).

[13] Wang, Y., Fang, Y., Lou, P., Yan, J. & Liu, N. Deep reinforcement learning based path planning for mobile robot in unknown environment. *J. Phys. Conf. Ser.* **1576**(1), 012009 (2020).

[14] Ibrahim, H. A., Azar, A. T., Ibrahim, Z. F. & Ammar, H. H. A hybrid deep learning based autonomous vehicle navigation and obstacles avoidance. In *Proceedings of the International Conference on Artificial Intelligence and Computer Vision (AICV2020)* 296–307 (Springer International Publishing, Cham, 2020).

[15] Ajeil, F. H., Ibraheem, I. K., Sahib, M. A. & Humaidi, A. J. Multi-objective path planning of an autonomous mobile robot using hybrid PSO-MFB optimization algorithm. *Appl. Soft Comput.* **89**, 106076 (2020).

[16] Gallage, M., Scaciota, R., Samarakoon, S., & Bennis, M. A simplified intelligent autonomous obstacle bypassing method for mobile robots. In *Proceedings of the 29th Annual International Conference on Mobile Computing and Networking* 1–3 (2023).

[17] Hao, X., Yin, L., Li, X., Zhang, L. & Yang, R. A Multi-objective semantic segmentation algorithm based on improved U-Net networks. *Remote Sens.* **15**(7), 1838 (2023).

[18] Yang, Y. Drone-view object detection based on the improved yolov5. In *2022 IEEE International Conference on Electrical Engineering, Big Data and Algorithms* (*EEBDA*) 612–617 (IEEE, 2022).

[19] Liu, F. & Qiu, S. Path planning of indoor mobile robot based on improved A* algorithm. In *2021 2nd International Conference on Artificial Intelligence and Information Systems* 1–4 (2021).

[20] Dai, X., Long, S., Zhang, Z. & Gong, D. Mobile robot path planning based on ant colony algorithm with A* heuristic method. *Front. Neurorobot.* **13**, 15 (2019). 31057388 10.3389/fnbot.2019.00015 PMC6477093

[21] Zheng, Y., Zeng, Q., Lv, C., Yu, H. & Ou, B. Mobile robot integrated navigation algorithm based on template matching VO/IMU/UWB. *IEEE Sens. J.* **21**(24), 27957–27966 (2021).

[22] Kwon, G. Y. & Shin, Y. J. Condition monitoring technique of HTS cable via tangent distance based template matching benefit. *IEEE Trans. Appl. Supercond.* **31**(5), 1–5 (2021).

[23] Li, P. Research on radar signal recognition based on automatic machine learning. *Neural Comput. Appl.* **32**(2), 1959–1969 (2020).

[24] Li, D., Deng, L. & Cai, Z. Design of traffic object recognition system based on machine learning. *Neural Comput. Appl.* **33**(14), 8143–8156 (2021).

[25] Ren, L., Wang, N., Pang, W., Li, Y. C. & Zhang, G. P. Modeling and monitoring the material removal rate of inferior belt grinding based on vision measurement and the gene expression programming (GEP) algorithm. *Int. J. Adv. Manuf. Technol.* **120**(1), 385–401 (2022).

[26] Xing, J., Wang, X. & Dong, J. Big data tracking and automatic measurement technology for unmanned aerial vehicle trajectory based on MEMS sensor. *Soft Comput.* **26**(9), 4237–4247 (2022).

[27] Tian, Y., Liu, H., Li, L., Yuan, G. J. & Wang, W. B. Automatic identification of multi type weld seam based on vision sensor with silhouette mapping. *IEEE Sens. J.* **21**(4), 5402–5412 (2021).

